# Disparities Exist in the Application of Low Tidal-volume Ventilation in the Emergency Department

**DOI:** 10.5811/westjem.59291

**Published:** 2023-04-26

**Authors:** Michael Self, Brent Kennis, Andrew Lafree, Christopher R. Tainter, Jesus Lopez, Atul Malhotra, Theodore Chan, Gabriel Wardi

**Affiliations:** * University of California San Diego, Department of Emergency Medicine, San Diego, California; † UC San Diego, Department of Anesthesiology, Division of Critical Care Anesthesiology, San Diego, California; ‡ UC San Diego, School of Medicine, San Diego, California; § UC San Diego, Department of Internal Medicine, Division of Pulmonary, Critical Care, Sleep Medicine & Physiology, San Diego, California

## Abstract

**Introduction:**

Low tidal-volume ventilation (LTVV), defined as a maximum tidal volume of 8 milliliters per kilogram (mL/kg) of ideal body weight, is a key component of lung protective ventilation. Although emergency department (ED) initiation of LTVV has been associated with improved outcomes, disparities in LTVV application exist. In this study our aim was to evaluate whether rates of LTVV are associated with demographic and physical characteristics in the ED.

**Methods:**

We conducted a retrospective observational cohort study using a dataset of patients who underwent mechanical ventilation at three EDs in two health systems from January 2016–June 2019. Demographic, mechanical ventilation, and outcome data including mortality and hospital-free days were abstracted by automatic query. A LTVV approach was defined as a tidal volume ≤8 mL/kg ideal body weight. We performed descriptive statistics and univariate analysis as indicated, and created a multivariate logistic regression model.

**Results:**

Of 1,029 patients included in the study, 79.5% received LTVV. Tidal volumes of 400–500 mL were used in 81.9% of patients. Approximately 18% of patients had tidal volumes changed in the ED. Female gender (adjusted odds ratio [aOR] 4.17, *P*< 0.001), obesity (aOR 2.27, *P*< 0.001), and first-quartile height (aOR 12.2, *P* < 0.001) were associated with receiving non-LTVV in multivariate regression analysis. Hispanic ethnicity and female gender were associated with first quartile height (68.5%, 43.7%, *P* < 0.001 for all). Hispanic ethnicity was associated with receiving non-LTVV in univariate analysis (40.8% vs 23.0%, *P* < 0.001). This relationship did not persist in sensitivity analysis controlling for height, weight, gender, and body mass index. Patients who received LTVV in the ED had 2.1 more hospital-free days compared to those who did not (*P* = 0.040). No difference in mortality was observed.

**Conclusion:**

Emergency physicians use a narrow range of initial tidal volumes that may not meet lung-protective ventilation goals, with few corrections. Female gender, obesity, and first-quartile height are independently associated with receiving non-LTVV in the ED. Using LTVV in the ED was associated with 2.1 fewer hospital-free days. If confirmed in future studies, these findings have important implications for achieving quality improvement and health equality.

## INTRODUCTION

More than 250,000 patients receive mechanical ventilation each year in emergency departments (ED) in the United States.^1^–^3^ Although potentially life-saving, mechanical ventilation may cause harm through volutrauma, barotrauma, and atelectrauma—collectively referred to as ventilator-induced lung injury (VILI).^4^ Adherence to lung-protective ventilation strategies mitigates these injuries and has been associated with improved patient-centered outcomes, including decreased mortality, for patients with and without acute respiratory distress syndrome (ARDS).^5^ In addition to appropriate positive end expiratory pressure (PEEP), an important component of a lung-protective strategy is adherence to low tidal-volume ventilation (LTVV), defined as a tidal volume ≤ 8 milliliters per kilogram (mL/kg) ideal body weight (IBW).^6^,^7^

Decades of data suggest that use of LTVV decreases mortality and VILI in patients undergoing mechanical ventilation in the intensive care unit (ICU).^8^ Recent data has demonstrated benefits to initiating LTVV in the ED, including decreased mortality, increased ventilator-free days, and reduced healthcare costs.^9^–^11^ Additionally, for patients with ARDS, or at risk for ARDS, convincing data shows that early adjustment of ventilator settings to target LTVV impacts outcomes.^12^,^13^ Changes to tidal volumes are infrequently made in the ED after initial ventilator settings are selected.^14^ Increased ED patient volumes over the past decade, coupled with rising inpatient critical care occupancy, have resulted in more critically ill patients boarding in the ED for longer times while undergoing mechanical ventilation, highlighting the importance of careful attention to ventilator settings in the ED.^15^–^18^

Various disparities in ED patient care have been described, including gender and racial differences in pain management, traumatic brain injury management, and emergency cardiology interventions.^19^–^21^ While adoption of LTVV has increased in the past decade, few investigations have evaluated demographic disparities in the use of LTVV in the ED.^10^,^14^ Several observational studies in the ICU have shown that female and obese patients are less likely to receive LTVV.^22^,^23^ Furthermore, outcome data from mechanically ventilated patients in the ICU has previously shown significant differences in survival based on race and ethnicity,^24^ as well as body mass index (BMI).^25^ A recent analysis found that women were less likely to receive LTVV in the ED compared to men, although this finding was limited by small sample size and single-center design.^26^ Given the importance of ED ventilator management, it is critical to identify patients at risk for receiving inappropriate tidal volumes, as this may directly impact survival and development of ARDS. Such data may help inform protocols designed to ensure appropriate ventilator settings for patients who receive mechanical ventilation in the ED, increase awareness among emergency physicians, and alleviate disparities in care that may impact patient-centered outcomes.

**Population Health Research Capsule**

What do we already know about this issue?

*Low tidal volume ventilation is associated with improved patient-centered outcomes in patients undergoing mechanical ventilation in the ED*.

What was the research question?

*Are rates of inappropriate high tidal volumes associated with demographic and physical patient characteristics?*

What was the major finding of the study?

*Female sex (OR 4.17, 95% CI 2.7–6.3) and short stature (OR 12.2, 95% CI 7.8–19.0) were associated with non-LTVV*.

How does this improve population health?

*Attention to initial selection of tidal volume in females and those with short stature may help emergency physicians mitigate this modifiable health disparity*.

In our study we aimed to evaluate whether rates of LTVV were associated with demographic and physical characteristics. Using a large dataset from multiple institutions, we hypothesized that female gender, height, and elevated BMI would be associated with lower use of LTVV in the ED.

## METHODS

### Study Design and Setting

We conducted a retrospective, multicenter, observational cohort study consisting of patients who received endotracheal intubation (ETI) and mechanical ventilation at three EDs across two healthcare systems from January 2016–June 2019. Our institutional review boards approved this study under a waiver of informed consent. This manuscript was prepared in accordance with STROBE guidelines.^27^ The urban ED is a regional safety-net hospital and has approximately 55,000 patient visits per year. The suburban ED is a tertiary referral center serving a population of patients who largely receive their primary and subspecialty care at the institution and has approximately 40,000 patient visits per year. The rural ED is in a high needs, primary care health professional shortage area near the US-Mexico border in a low-density, agricultural community with approximately 50,000 patient visits per year. The urban and suburban sites are part of an academic health system, while the rural site is a distinct health system. Data from the urban and suburban sites was abstracted by automated query, while data from the rural site was extracted manually by two trained reviewers using a standardized data collection template.

### Selection of Participants

Patients who underwent ETI were identified by the presence of an intubation order, neuromuscular blockade order, mechanical ventilation order, or documentation of ETI within the study period. Identified patients were included in the study if they were at least 18 years of age, with fraction of inspired oxygen (FiO2) and PEEP values recorded in the ED and with complete weight, height, and demographic data, and a height greater than 1.52 meters (the lower limit of acceptable height when calculating IBW using the Devine formula). We excluded patients with implausible data (eg, FiO2 > 1.00 or < 0.21). Missing data was not substituted for any cases.

### Mechanical Ventilation

At both health systems, initial mechanical ventilation settings are decided upon by the treating physician and entered by a respiratory therapist. At the urban and suburban EDs, a hospital-wide protocol sets the default ventilator mode to volume-targeted pressure control (VTPC). The treating physician may choose a different mode of ventilation, but virtually all patients receive VTPC. At the rural hospital, initial ventilator settings are input by the treating physician. If an initial order for mechanical ventilation settings is not placed, a respiratory-therapist protocol allows selection of initial mechanical ventilation settings with volume control, PEEP of 8, and respiratory rate set based on the patient’s clinical picture. Measuring patient height with a tape measure is recommended but not mandated in both healthcare systems.

### Measurements

Baseline demographics (including height, weight, gender, race, ethnicity, and comorbidities), vital signs, laboratory values, ventilator settings, maximum sequential organ failure assessment (SOFA) score within 24 hours, hospital discharge disposition, ED length of stay, and ED boarding time were abstracted by automated query from the electronic health record (EHR). Height and weight were obtained by any of the following means: directly measured in the ED; patient reported; obtained from patient identification; or obtained from prior ED visits. Gender was recorded as assigned gender at birth in the EHR and was patient reported, obtained from previous visits, or obtained from patient identification.

We retrospectively collected race and ethnicity from the EHR in accordance with recent guidance on appropriate reporting of race and ethnicity in scientific and medical journals.^28^ Registration staff record race and ethnicity data through protocolized approaches to obtain self-reported race and ethnicity from the patient or patient surrogate, if the patient is unable to provide information. The EHR’s prespecified race and ethnicity categories, based on nationally accepted categories, are reported as more detailed information but was not available. “Other Race or Mixed Race” and “Unknown” are reported with quotation marks as these are not specific races but EHR constructs. Vital signs and ventilator settings were abstracted from nurse or respiratory therapist-verified flowsheets, respectively. The SOFA scores were automatically calculated by the EHR. Our institution only started automatically calculating SOFA scores in July 2017; thus, a number of patients did not have this score recorded. The Charlson Comorbidity Index was calculated as described by Charlson et al.^29^ Ideal body weight was calculated based on patient height using the Devine formula.^30^ We abstracted BMI from the chart, and obesity was defined as a BMI >30 kilograms per meter squared (kg/m^2^). Hospital-free days were defined as days out of the hospital in a 28-day period and were calculated using the date and time of ED arrival and hospital disposition. A patient who expired during admission received zero HFDs.

### Outcome Measures

The primary outcome was the use of tidal volume (Vt) > 8 mL/kg IBW with mechanical ventilation in the ED. Secondary outcomes included the percentage of patients who had a tidal volume change in the ED, hospital-free days, and mortality.

### Analysis

We used descriptive statistics and frequency distributions to compare patient characteristics. Categorical variables were compared using the chi-squared and Fisher exact test where appropriate. We compared continuous variables using the independent two-sample *t*-test or the Mann-Whitney U test as indicated. Data normality was assessed by examining kurtosis and skewness and inspection of histograms.

We developed a logistic regression model to analyze the relationship between receiving Vt > 8 mL/kg IBW and baseline patient demographics. A priori variables of known significance to the outcome (female gender, BMI >30 kg/m^2^) and clinically relevant and biologically plausible variables (age, first-quartile height) were included in the model. We used a multivariate backwards stepwise logistic regression model that selected variables sequentially for inclusion or exclusion at the 0.10 significance level. The model’s goodness-of-fit was assessed by the Hosmer-Lemeshow test and R-squared values. We reported adjusted odds ratios (aOR) and corresponding 95% CIs for all variables in the model. All tests used a two-tailed approach, and a *P*-value of < 0.05 was considered significant. A second multivariate analysis was performed using the same methodology, with height, weight, female gender, age and Hispanic ethnicity, with variables selected based on biologic plausibility or results of univariate analysis.

## RESULTS

### Patient Demographics

**Figure 1:**
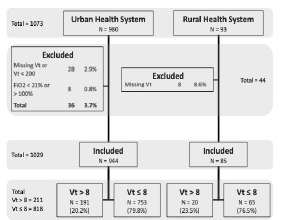
Patient selection and exclusion. Tidal volume (Vt) > 8: tidal volume > 8 mL/kg ideal body weight. Vt ≤ 8: tidal volume ≤ 8 mL/kg ideal body weight. *ED*, emergency department; *MV*, mechanical ventilation; *Vt*, tidal volume, *FiO_2_*, fraction of inspired oxygen.

We assessed a total of 1,073 cases for inclusion from the automatic query. Of these, 1,029 were included in the final study population. Patients were excluded for initial FiO2 <0.21 or >1.00 (8, 0.75%) (Figure 1). Data regarding length of stay and mortality was missing for 94 (9.14%) patients, primarily from the rural site, and were excluded from calculations of hospital-free days and mortality. Baseline characteristics are shown in Table 1. The mean age was 56 years, 31.2% of patients were female, 54.9% were identified as White, 13.5% were identified as Black, and 4.5% were identified as Asian. A total of 25.0% were identified as “Other Race or Mixed Race” patients. Of these, 73.9% were identified as having Hispanic ethnicity, compared to 14.2% of White patients and 23.5% of “Unknown” patients (*P* <0.001). A total of 26.7% were identified as Hispanic patients. The median BMI was 25.9 (range 22.4–30.5), and 27.1% had a BMI > 30 kg/m^2^. A total of 315 (30.6%) patients were in the first quartile of height (1.52–1.65 meters).

### Intubation Indication, Initial Ventilator Settings and Modifications in the Emergency Department

Airway protection was the most common reason for intubation (44.1%), followed by primary respiratory failure (31.5%), cardiac arrest (12.6%), and refractory shock (11.5%). There was no clinically or statistically significant difference in intubation indication between patients who received Vt > 8 mL/kg IBW and those who did not (*P* = 0.91), nor was there a difference in Vt/IBW (*P* = 0.40). Initial ventilator settings in the ED, as well as changes to those settings while the patient remained in the ED are provided in Table 2. Most patients (65.5%) had an initial Vt between 6–8 mL/kg IBW, while 148 (14.0%) had an initial Vt < 6 mL/kg IBW, and 211 (20.5%) had an initial Vt > 8 mL/kg IBW. Initial tidal volume was most frequently set at either 500 mL (36.5%) or 450 mL (28.2%). Tidal volume was changed in the ED in 183 patients (17.8%). Only 4% of ventilator changes in the ED corrected non-LTVV to LTVV.

### Low Tidal Volume Ventilation and Sex, Race and Ethnicity

A total of 211 patients (20.5%) had an initial Vt set at > 8 mL/kg IBW (Table 1). Patients who received Vt > 8 mL/kg IBW were more frequently female than male (75.8% vs 19.7%, *P* < 0.001). Females also had a significantly higher Vt/IBW than males (8.0 +/− 1.14 mL/kg vs 6.7 +/− 0.89 mL/kg, *P* < 0.001). Race was found to be significantly associated with Vt > 8 mL/kg IBW (*P* < 0.001). Specifically, patients who received Vt > 8 mL/kg IBW were more frequently identified as “Other or Mixed Race” (36.5% vs. 22.0%, *P* < 0.001), while those who received Vt ≤ 8 mL/kg IBW were more frequently identified as White (56.6% vs 48.3%, *P* < 0.001) or Black (14.9% vs 8.1%, *P* **=** 0.009), when compared against all other racial categories. Patients who received Vt > 8 mL/kg IBW were also more likely to be Hispanic (40.8% vs 23.0%, *P* < 0.001) as compared to non-Hispanic patients. Vt/IBW followed a similar trend, with a significant difference found between “Other or Mixed Race” patients and both Black and White patients (7.4 +/− 1.3 vs. 6.8 +/− 1.0 and 7.0 +/− 1.1, respectively, *P* < 0.001) as well as Hispanic patients and non-Hispanic patients (7.4 +/− 1.3 vs 7.0 +/− 1.1, *P* < 0.001).

### Low-tidal Volume Ventilation and Body Mass Index, Height Quartile, Comorbidities, and Maximum SOFA Score

Obese patients (BMI >/= 30 kg/m^2^) were more likely to receive Vt > 8 mL/kg IBW (40.8% vs 23.6%, *P* < 0.001) and had a higher Vt/IBW than patients without obesity (7.5 +/− 1.1 mL/kg vs 7.0 +/− 1.1 mL/kg, *P* < 0.001). Patients in the first quartile for height were more likely to receive Vt > 8 mL/kg IBW (82.5% vs 17.2%, *P* < 0.001) and had a higher Vt/IBW than patients in other quartiles (8.1 +/− 1.2 mL/kg vs 6.7 +/− 0.81 mL/kg, *P* <0.001). Patients in all other quartiles were more likely to receive Vt ≤ 8 mL/kg IBW (*P* < 0.001 for all). Female, Hispanic, “Other or Mixed Race” and “Unknown race” patients more frequently had a first quartile height than male gender, and other ethnicities and races (68.5%, 43.7%, 41.2%, 52.9%, respectively; *P* < 0.001 for all). Patients who received Vt > 8 mL/kg IBW were more likely to have diabetes (35.5% vs 26.1%, *P* **=** 0.01); otherwise, there were no differences between the groups. The mean maximum SOFA score of 9 was the same between the groups and overall cohort.

### Low-tidal Volume Ventilation and Outcomes

There was no difference in hospital mortality for patients who received at least 48 hours of mechanical ventilation with high and low tidal volume volumes (9.4% vs 11.8%, *P* = 0.40), nor was there a significant difference in Vt/IBW between patients who died compared to those who survived (7.0 +/− 1.2 mL/kg vs 7.1 +/− 1.1 mL/kg, *P* **=** 0.21). However, there was a significant reduction in hospital-free days for patients who received LTVV compared to those who did not (8.9 days vs 11.0 days, *P* **=** 0.03).

### Multivariate Regression Analysis

Age, female gender, BMI > 30 kg/m^2^ and first-quartile height were included a priori in the multivariate regression analysis. Table 3 shows the results of the multivariate regression analysis.

First-quartile height and female gender were strongly associated with receiving Vt > 8 (aOR 12.2, *P* < 0.001 and aOR 4.17, *P* < 0.001, respectively). BMI >30 kg/m^2^ (aOR 2.27, *P* < 0.001) was also independently associated with receiving Vt > 8. Age was not significantly associated with receiving Vt > 8 mL/kg IBW. These associations persisted in second multivariate analysis performed, with strong associations between height, weight, female gender, and receiving Vt > 8 mL/kg IBW. There was no association between Hispanic ethnicity and Vt > 8 mL/kg IBW in this second analysis.

## DISCUSSION

Our analysis is the largest to date showing that female gender, first quartile height, and obesity are independently associated with receiving Vt > 8 mL/kg IBW in ED patients. Also, we showed that physicians use a narrow range of convenient, round tidal volumes (450 mL, 500 mL) with overall poor correlation to anthropomorphic characteristics such as height, weight, and BMI. Although this practice resulted in over 80% of patients receiving LTVV, we found that female gender and Hispanic ethnicity were factors associated with receiving non-LTVV, and these patients were more often in the first quartile for height. Finally, we found that these ED tidal volume disparities and practice patterns were associated with 2.1 fewer hospital-free days.

We speculate that emergency physicians infrequently use a height-based calculation for tidal volume, rather using a narrow range of seemingly preset volumes. Tidal volumes of either 400, 450, or 500 mL were used in 81.9% of patients. Only 6.2% of patients received a Vt < 400 mL, which represents the upper limit of the LTVV goal for a man 60 inches tall or a woman 62 inches tall. This could in part explain why the 13.1% of our cohort that was <62 inches was significantly less likely to receive LTVV. While Wiess et al made similar observations and hypotheses in their 2016 study of ICU patients, ours extends this observation to ED patients.^10^,^31^ That the shortest patients in our cohort received the highest Vt/IBW fits with this conclusion.

Furthermore, patients were unlikely to have tidal volume adjusted in the ED, with only 18% of patients having a change. Of patients with a Vt change, 22% were changed from a non-LTVV to LTVV and 12% were changed from LTVV to non-LTVV. While Vt changes away from LTVV appear concerning, there are several plausible reasons to make such an adjustment that our study did not account for (ie, severe acidosis requiring higher minute ventilation). More importantly, our study shows that few ED patients will receive a Vt adjustment, emphasizing the importance of appropriate initial ventilator settings. Furthermore, it is imperative to remember that height based Vts are a convenient initial estimate that must be fine-tuned based on the individual patients’ pulmonary mechanics, the management of which is outside the scope of this work.

Importantly, we demonstrated that this practice pattern results in female and Hispanic patients, who were more often in the first quartile for height, receiving non-LTVV more frequently, an important disparity given that non-LTVV was associated with fewer hospital-free days. We found no association between Hispanic ethnicity and Vt > 8 mL/kg IBW when controlling for height and gender in a sensitivity analysis, supporting this conclusion. Although the “Other or Mixed Race” racial category was also associated with non-LTVV we suspect that there is significant overlap between this grouping and Hispanic ethnicity. Over 74% of “Other or Mixed Race” patients also identified as Hispanic, significantly more than any other racial group, and these groups had an identical Vt/IBW. While multiple studies have demonstrated disparities in topics ranging from analgesic practices, intensity of ICU care, and outcomes from critical illness, ours is the first to our knowledge to do so with respect to LTVV in the ED.^32^,^33^ Unlike many healthcare disparities with complicated origins, a simple change in practice pattern from “preset” tidal volumes to a calculated, height-based tidal volume could mitigate this issue.

While first-quartile height had the strongest association, the presence of obesity and female sex were also independently associated with non-LTVV. We hypothesize that obesity may cause physicians to overestimate patients’ tidal volume, supported by the observation that obesity is associated with a higher Vt/IBW compared to non-obese patients. We also suspect that female gender led physicians to overestimate patients’ tidal volume demands for reasons beyond the association between female gender and first-quartile height. Commonly used models for the prediction of IBW estimate lower weights for women than for men of the same height.^30^ It is possible that this consideration is underappreciated when estimating tidal volume requirements. Similarly, we suspect that the tidal volume demand of patients of first-quartile height was frequently overestimated. It has previously been shown that clinicians perform poorly at estimating the height of patients.^34^

The patients in our cohort who did not receive LTVV had 2.1 fewer hospital-free days than those who received LTVV (8.9 vs 11.0, *P* = 0.04). This observation is consistent with several recent studies showing that initiation of LTVV as part of a lung-protective strategy in the ED is associated with better patient outcomes. While we did not find a significant difference in mortality, prior studies utilizing LTVV as part of a multifaceted lung protective ventilation strategy have done so when adjusting for comorbidities and illness severity.^9^–^11^ While the reason for this is uncertain, there are several possibilities. First is that we assessed a single component of a lung-protective ventilation strategy and did not analyze PEEP or other ventilator settings included in other ED-based investigations, such as the LOV-ED trial by Fuller et al.^10^ Second, we did not include severity of illness as a covariate in our analysis due to insufficient data. Third, although the data supporting the use of LTVV is strongest for patients with or at risk for ARDS, we did not include presence of ARDS due to insufficient data. Despite this, our analysis shows that the demographic variability in the application of LTVV has meaningful clinical consequences and thus warrants intervention.

We believe that the patient characteristics identified in our analysis as risk factors for inappropriately high tidal volumes can serve as targets for improvement. A 2021 trial by Tallman et al showed that providing respiratory therapists with a tape measure increased the rate of LTVV in the ED/ICU patients.^35^ This low-cost, practical intervention would allow measured height to guide Vt, rather than estimated height or absolute body weight. Finally, we hope that awareness of the trend that certain demographics are less likely to receive LTVV will contribute to the collective cognitive effort to reduce healthcare disparities.

## LIMITATIONS

Our study has several limitations. With a retrospective design, our study could only show associations and was vulnerable to incomplete and inaccurate documentation. Mortality and hospital-free days data was missing from approximately 9% of patients and was excluded from those calculations, which may have affected outcomes data. Race and ethnicity data was abstracted from the EHR and the source of this information was not available, making it subject to reporting bias, although both institutions place major emphasis on self-reported demographic information. The Pew Research Center has shown that Hispanic patients are often confused by or do not relate to the survey category of “White, Hispanic,” and instead identify as “other or mixed.”^36^ Accordingly, a significant association was detected between Hispanic ethnicity and other/mixed race in our population. Due to limitations in our retrospective dataset, we did not include the presence of or risk factors for ARDS, which may have influenced outcome data.

## CONCLUSION

Female gender, obesity (body mass index > 30), and first-quartile height are independently associated with receiving non-low-tidal volume ventilation in the ED. Emergency physicians use a narrow range of default initial tidal volumes that may not meet lung-protective ventilation goals for many patients, with few corrections. Future prospective studies are required to validate these findings.

## Figures and Tables

**Table 1:** Study population characteristics.

	Total N = 1,029	Initial Vt > 8 mL/kg IBW n = 211 (20.5%)	Initial Vt ≤ 8 mL/kg IBW n = 818 (79.5%)
Age, y	56.3 (17.2)	57.9 (18.2)	55.9 (16.8)
Female, No. (%)	321 (31.2)	160 (75.8)	161 (19.7)
Males, No. (%)	708 (68.8)	51 (24.2)	657 (80.3)
Race, No. (%)			
Black	139 (13.5)	17 (8.1)	122 (14.9)
Asian	46 (4.5)	9 (4.3)	37 (4.5)
Native American	2 (0.2)	0 (0.0)	2 (0.2)
Pacific Islander	3 (0.3)	1 (0.5)	2 (0.2)
Other or mixed	257 (25.0)	77 (36.5)	180 (22.0)
Unknown	17 (1.7)	5 (2.4)	12 (1.5)
White	565 (54.9)	102 (48.3)	463 (56.6)
Hispanic, No. (%)	274 (26.7)	86 (40.8)	188 (23.0)
Weight (kg)	77.0 (65.7 – 91.0)	74.1 (63.4 – 87.2)	77.7 (56.0 – 92.0)
BMI (kg/m2)	25.9 (22.4 – 30.5)	25.2 (21.9 – 29.6)	28.4 (24.8 – 33.9)
BMI > 30, No. (%)	279 (27.1)	86 (40.8)	193 (23.6)
Height quartile, No. (%)			
1st (min. – 1.65 m)	315 (30.6)	174 (82.5)	141 (17.2)
2nd (1.66 m – 1.73 m)	258 (25.1)	28 (13.3)	230 (28.1)
3rd (1.74 m – 1.80 m)	253 (24.6)	8 (3.8)	245 (30.0)
4th (1.81 m – max.)	203 (19.7)	1 (0.5)	202 (24.7)
Rural hospital, No. (%)	85 (8.3)	20 (9.5)	65 (7.9)
Urban safety-net hospital, No. (%)	825 (80.1)	161 (76.3)	664 (81.2)
Urban academic center, No. (%)	119 (11.6)	30 (14.2)	89 (10.9)
Indication for intubation, No. (%)			
Airway protection	454 (44.1)	93 (44.1)	361 (44.1)
Cardiac arrest	130 (12.6)	29 (13.7)	101 (12.3)
Primary respiratory failure	324 (31.5)	63 (29.9)	261 (31.9)
Refractory shock	121 (11.8)	26 (12.3)	95 (11.6)
CCI Score (n = 915)	3 (1 – 6)	3 (1 – 6)	3 (1 – 6)
Comorbidities, No. (%)	n = 915	n = 183	n = 732
Cancer	148 (16.2)	27 (14.8)	121 (16.5)
Cerebrovascular disease	216 (23.6)	48 (26.2)	168 (23.0)
CHF	262 (28.6)	53 (29.0)	209 (28.6)
Chronic lung disease	292 (31.9)	62 (33.9)	230 (31.4)
Diabetes mellitus	256 (28.0)	65 (35.5)	191 (26.1)
HIV	29 (3.2)	3 (1.6)	26 (3.6)
Liver disease	219 (23.9	37 (20.2)	182 (24.9)
Myocardial infarction	117 (12.8)	22 (12.0)	95 (13.0)
Renal disease	195 (21.3)	38 (20.8)	157 (21.4)
Max. SOFA score (n = 652)	9 (4)	9 (4)	9 (4)
Length of stay (min.; n = 935)	313 (234 – 468)	313 (215 – 484)	314 (236 – 461)
Boarding time (min.; n = 935)	139 (86 – 229)	134 (90 – 232)	140 (85 – 228)
Mortality, admitted ≥48 hrs, No. (%)	116 (23.4)	20 (9.4%)	96 (11.8%)
Hospital-free days (d.; n = 904)	10.4 (15.3)	8.9 (±11.4)	11.0 (±16.6)

Notes: data are reported as mean (SD), median (IQR) or number (%).

*mL*, milliliter; *kg/M^2^*, kilogram, meter squared; *Vt*, tidal volume; *IBW*, ideal body weight; *BMI*, body mass index; *CHF*, congestive heart failure; *SOFA*, sequential organ failure assessment; *CCI*, Charlson Comorbidity Index; *m*, meter.

**Table 2:** Initial tidal volume settings and changes.

Variable (N = 1,029)	No. (%)
Initial Vt, range	270 – 700
Initial Vt 600 mL	22 (2.1)
Initial Vt 550 mL	56 (5.4)
Initial Vt 500 mL	376 (36.5)
Initial Vt 450 mL	290 (28.2)
Initial Vt 400 mL	177 (17.2)
Initial Vt 350 mL	36 (3.5)
Vt Changed in ED	183 (17.8)
Initial Vt 6–8 mL/kg IBW	674 (65.5)
Initial Vt < 6 mL/kg IBW	144 (14.0)
Initial Vt >8 mL/kg IBW	191 (18.6)
Initial Vt >10 mL/kg IBW	20 (1.9)

*IBW*, ideal body weight; *Vt*, tidal volume; *mL*, mililiter; *kg*, kilogram; *ED*, emergency department.

**Table 3:** Multivariate regression analysis results (N=1,029).

Variable	aOR	95% CI	SE	P-value
Age	0.997	0.986 – 1.01	0.01	0.56
Female sex	4.17	2.73 – 6.36	0.22	< 0.001
BMI >30	2.27	1.49 – 3.47	0.22	< 0.001
1st quartile height	12.2	7.81 – 19.0	0.23	< 0.001

*aOR*, adjusted odds ratio; *SE*, standard error; *BMI*, body mass index; *ED*, emergency department.
